# Effects of sample pre-treatments on the analysis of liquid organic manures by visible and near-infrared spectrometry

**DOI:** 10.1016/j.heliyon.2024.e27136

**Published:** 2024-02-27

**Authors:** Michael Horf, Robin Gebbers, Hans-Werner Olfs, Sebastian Vogel

**Affiliations:** a Leibniz Institute for Agricultural Engineering and Bioeconomy (ATB), Department for Agromechatronics, Max-Eyth-Allee 100, 14469 Potsdam, Germany; b Martin Luther University Halle-Wittenberg, Chair of Farm Management, Karl-Freiherr-von-Fritsch-Straße 4, 06120 Halle, Germany; c University of Applied Sciences, Faculty of Agricultural Science and Landscape Architecture, Chair of Plant Nutrition and Crop Production, Am Krümpel 31, 49090 Osnabrück, Germany

**Keywords:** Biogas digestates, Centrifuging, Drying, Filtration, Optical spectroscopy, Livestock manure, Climate change

## Abstract

Proper application of a fertilizer requires precise knowledge of its nutrient composition. In the case of liquid organic manures (LOM), this information is often lacking due to heterogeneous nature of these fertilizers. Published “book values” of nutrient contents present the average from a wide range of possible nutrient characteristics, but usually differ considerably from the concentration in a particular manure. Thus, chemical analyses are recommended before applying the specific LOM. Unfortunately, this is usually too costly and time-intensive in practical farming. On-farm analysis by optical spectrometry in the visible and near-infrared (Vis-NIR) range is considered as an efficient alternative. However, calibration of Vis-NIR spectrometry for LOM is challenging as shown in many studies. One reason is LOMs’ tendency to rapidly segregate into a fuzzy continuum with liquid and solid properties. By separating LOM into well-defined liquid and solid phases and measuring them separately, calibration of Vis-NIR spectrometry might be improved. In this study, the effects of four sample pre-treatment techniques on the prediction accuracy of macronutrients (N, P, K, Mg, Ca, S), micronutrients (B, Mn, Fe, Cu, Zn), dry matter and pH of LOM using visible and near infrared spectrometry were comprehensively investigated. The concentrations were referred either to wet basis or to dry matter basis. For the study, a total of 163 samples, separated in two similar LOM sets (pig, cattle, digestates), were either dried, filtered, or centrifuged and always compared to non-treated samples. The experiments demonstrate that in comparison to raw samples (Ø r^2^ = 0.85) neither filtering (Ø r^2^ = 0.76 for filtrates and Ø r^2^ = 0.71 for filter residues), centrifugation (Ø r^2^ = 0.59 for supernatants and Ø r^2^ = 0.79 for pellets), nor drying (Ø r^2^ = 0.74) revealed to be a helpful preparation step significantly improving prediction results, independent from referring to wet or dry basis concentrations.

## 1 Introduction

Recently, the availability of mineral fertilizers has become increasingly limited due to growing energy prices, progressive depletion of mineral raw materials and the disruption of supply chains as a consequence of political and social crises [1]. This may rise uncertainties in agricultural production and lower food security worldwide. Thus, there is a growing demand for organic fertilizers as supplementary or fundamental alternative including more sustainable production chains. The replacement of conventional mineral fertilizers by a demand-oriented fertilization with liquid organic manures (LOM), including biogas digestates, holds great potential for saving both raw materials and energy as well as for sustaining agricultural production [[2], [3], [4]]. Furthermore, the energy intensive production of mineral fertilizers, especially of synthetic nitrogen, has a strongly negative impact on climate change through a significant amount of global greenhouse gas (GHG) emissions (2.1 % of total global GHG in 2018 [5]), which is more than the potential GHG emissions of prudently applied LOM [6,7]. In contrast, LOM can be considered a valuable, low-cost and regionally abundant resource with a beneficial impact on soil health [8]. LOM mainly consist of animal excreta, i.e. urine and faeces, as well as bedding materials, feed residues, and water, and in case of biogas digestates of fermented plant residuals. It is estimated that livestock farming and biogas production generate more than one billion tons of LOM per year in both the European Union [9] and the United States of America [10]. These LOM contain all essential macro- and micronutrients required for a healthy plant growth [11]. The precise knowledge of these nutrient concentrations in LOM is a prerequisite for an optimal crop supply and for avoiding environmental problems caused by overfertilization [12]. However, the inherent heterogeneity of LOM makes it challenging to exactly quantify the amounts of nutrients applied to agricultural land [13].

Generally, farmers have four options for estimating the nutrient concentration in LOM: (i) consulting tabulated values from issued literature (book values), (ii) dispatching representative samples to a laboratory for a the standardized chemical analysis, (iii) implementing on-farm quick test methods, or (iv) just trusting in personal experience, which is probably the main reason for overfertilization. Book values, as provided by various organizations [14], are also frequently used by farmers since this is easy, fast and inexpensive. However, tabulated values cannot precisely predict the nutrient concentrations of a particular LOM since it depends on several factors, e.g. animal species and age, feedstuff compositions, water content, homogenization technique, storage management, climate factors etc. [15]. On the other hand, standard laboratory analyses take a lot of time, cause costs and chemical waste, and depend on the quality of a representative sampling [16]. Taking a representative sample is not a simple procedure as many LOM segregate into a fuzzy continuum from more liquid to more solid phases. In pig LOM, the solids sink to the bottom of the storage tank, whereas in dairy LOM, the solids float on top of the liquid phase. Furthermore, the solid and liquid phase comprise different nutrient compositions. According to Saeys et al. [17] the liquid phase contains more dissolved potassium (K^+^) and ammonium (NH_4_^+^), whereas the solid phase contains more organic matter including organically bound phosphorous (P), calcium (Ca), and magnesium (Mg). Before sampling, remixing LOM in a storage tank for homogenization is technically demanding and increases harmful ammonia (NH_3_) and hydrogen sulphide (H_2_S) emissions [18]. As a third option for a quantification of LOM nutrients on-farm quick tests can be used for the analysis of some parameters [19,20]. However, the quality of on-farm quick tests also depends on representative sampling.

In order to comply with regional regulations for LOM applications, e.g. EU limit for nitrogen: 170 kg per ha and year (EU regulation 2092/91), or EU limit for nitrate: 50 mg/L in ground-, surface- and drinking water (European Commission directive 91/676/EEC), surplus LOM needs to be transported from intensive livestock regions to regions with arable farming. However, since the exact nutrient composition is unknown, LOM is often treated as a waste material rather than a useful fertilizer product. Therefore, an accurate nutrient analysis would contribute to a positive perception of LOM as a value-added fertilizer by arable farmers and as a profitable product in livestock farming. This would be an important contribution towards a closure of nutrient cycles in agriculture and the establishment of a circular bioeconomy [21].

For these reasons, there is a high demand for alternative and practical technologies, which quantify the composition of LOM rapidly, reliably, and at low costs. Optical spectrometry in the visible and near-infrared range (Vis-NIR, 350–2500 nm) has the potential to meet these requirements. Due to its suitability for on-farm and on-line analysis, it may become an important addition to or even replace the currently applied measurement methods if it can be offered at affordable prices and guarantees sufficient accuracy, whether it is measured in the storage tank, while filling a slurry tanker, or during application in the field [22].

The rapid development of chemometrics during the last three decades [23] has promoted Vis-NIR spectroscopy as a fast, clean and non-destructive measuring technology in many applications. With regard to manure analysis, Asai et al. [24] were one of the first who published a study using spectrometry in the near infrared region to predict total carbon (TC) and total nitrogen (TN) in dried dairy manure. Since then, over 40 articles were published on the analysis of liquid, dried, or composted manures and biogas digestates by visible (Vis), near infrared (NIR) or mid infrared (MIR) spectrometry ([25] and references therein). During the last years, even on-line measurements of LOM at farm and field scale were successfully tested for total nitrogen (TN), ammonium-N (NH_4_–N), phosphorous (P), potassium (K), and dry matter (DM) content [26,27].

However, detailed studies are missing regarding the effects of different sample pre-treatment techniques like filtering, centrifuging or drying on the prediction performance using optical spectrometry for LOM samples representing the content of a whole slurry tank. Although in some publications, dried organic manures were analysed [[28], [29], [30], [31]], the authors did not compare them with analyses of the original liquid samples. In Finzi et al. [32] a filtering technique was compared with untreated manure. However, they exclusively analysed the filtrate and not the filter residue.

For these reasons, this research has the potential to fill a gap in the existing literature. The objectives of the present Vis-NIR study were

(i) to examine the effects of four different pre-treatment techniques of LOM samples (untreated, filtering, centrifuging, and drying) on the prediction accuracy of nutrients (TN, NH_4_–N, P, K, Mg, Ca, sulphur (S), manganese (Mn), iron (Fe), copper (Cu), zinc (Zn), and boron (B)), as well as DM and pH in pig, cattle, and digestate LOM, and
(ii) to compare chemometric predictions referring to either wet or dry basis concentrations.

## 2 Materials and methods

### 2.1 Sample sets

Due to the available sample quantities, sample preparation procedures were conducted with two different but very similar samples sets. Set 1 consisted of 62 samples and set 2 of 111 samples from liquid livestock manures and biogas digestates. The samples were collected on several farms in Northwest Germany between 2018 and 2020. Each sample was homogenised with a stainless-steel mixer (Blender CB15VXE, Waring Commercial, USA) before subsampling for reference analyses and spectral measurements. Samples were stored at −18 °C in 500 ml plastic bottles.

In order to assess possible effects of different LOM types, the evaluations were carried out for the entire sample set as well as for the subgroup "pig" and in set 2 additionally for the subgroup "cattle” LOM. No separate evaluation could be performed for biogas digestates due to the limited number of samples.

### 2.2 Reference analyses

The reference analyses were conducted at LUFA Nord-West (Hameln, Germany), a certified laboratory for slurry analysis, by applying European normed standard laboratory methods. The following 14 parameters were determined: dry matter (DM), pH, total nitrogen (TN), ammonium nitrogen (NH_4_–N), phosphorous (P_2_O_5_), potassium (K), calcium (Ca), magnesium (Mg), sulphur (S), as well as the micronutrients boron (B), copper (Cu), manganese (Mn), iron (Fe), copper (Cu), and zinc (Zn). For more detailed information on the laboratory analyses, see Ref. [22]. The laboratory measurement uncertainty concerning the repeatability was between 1.7 and 3.6 % for all target parameters (LUFA-Nord-West; personal communication). All element concentrations were either expressed on a wet or dry weight basis and both later used in the chemometric analyses to examine potential differences on the prediction accuracy.

### 2.3 Sample pre-treatments and subsample sets

The aim of the various sample preparation procedures was to separate the liquid phase from the solid phase and to analyse them separately. Therefore, the spectrometric analyses of each material obtained by drying, filtering, or centrifuging were compared to those of original, unseparated samples. Prior to spectral analysis, the frozen samples were thawed over night at room temperature. After thorough shaking, the samples were treated by one of the following sample pre-treatment procedures.

• set 1 & 2: no sample preparation (∼25 ml), i.e. original (raw) LOM samples as reference
• set 1: vacuum filtration using a water jet pump and a 100 μm nylon filter in a Buchner funnel (∼100 ml)
• set 1: drying at 65 °C and subsequent grinding (∼60 ml)
• set 2: centrifuging at 2500 revolutions per second for 15 min at 15 °C (∼50 ml)

These four sample pre-treatment steps resulted in seven different subsample sets.

• set 1a: untreated (raw) LOM for comparing with drying and filtration effects
• set 1b: filter residues
• set 1c: filtrate
• set 1d: dried LOM
• set 2a: untreated (raw) LOM for comparing with centrifugation effects
• set 2b: centrifuge pellets
• set 2c: centrifuge supernatants

### 2.4 Spectral measurements

Spectral measurements were conducted with the ultra-broadband UV–Vis–NIR spectrometer ARCspectro UV–Vis–NIR FIB (ARCoptix S.A., Switzerland) using a wavelength range of 325–2450 nm. It combines a diode array detector for the UV–Vis range with a Fourier transform detector for the NIR range. The spectral resolution (expressed as full width at half maximum; FWHM) is < 5 nm and the reporting interval is 1 nm, resulting in a total of 2125 predictor variables. For more details, see Ref. [22].

For the spectral analysis, about 25 ml of liquid material (original LOM, filtrates, centrifuge supernatants) or 10 g of solid material (dried LOM, filter residues, centrifuge pellets) were filled in glass vials (2.8 mm diameter, thin borosilicate glass with a planar bottom; Deutsche Metrohm, Germany). Immediately before measurements, liquid samples were intensively stirred. During spectral analysis, samples were illuminated by two 50 W tungsten halogen lamps at an angle of 45° (Fig. 1). Diffuse reflected light from the bottom of the sample vials was transmitted to the spectrometer via two glass fibres for the Vis and NIR range. Measurements were conducted at four positions by rotating the glass vial by 90°. At each position, a spectrum was derived by averaging 50 replicates in the UV–Vis range with an integration time of 60 ms and 16 replicates in the NIR range using the medium gain factor. Afterwards, the four obtained spectra were averaged.

**Fig. 1:**
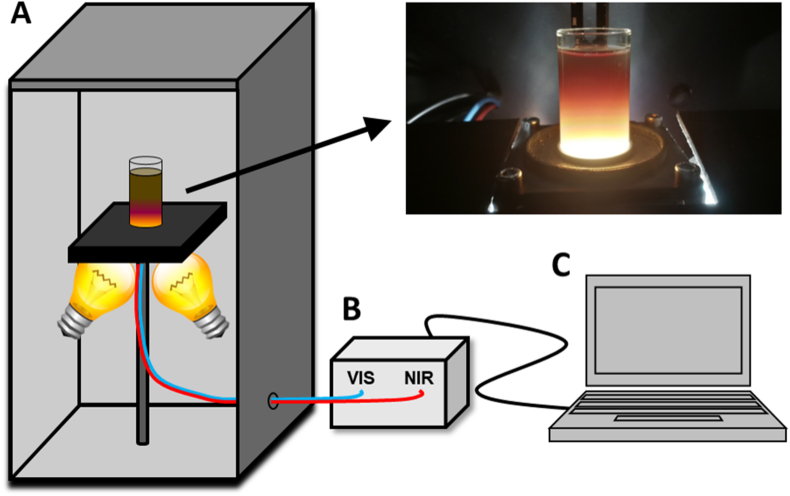
Setup for spectroscopic measurements of pre-treated liquid organic manures (LOM). A) Inside a box, protected from ambient light, two 50W tungsten halogen lamps illuminate a glass vial with a planar bottom. The glass vial is filled with sample material. Two optic fibres for the Vis and NIR range transmit the reflected light of the sample to B) a UV–Vis–NIR spectrometer, which is connected to C) a computer. Adapted from Ref. [16].

As a durable, heat-resistant in-house reflection standard, a dark mat ceramic plate was manufactured and referenced to a certified 10% diffuse PTFE reflection standard (Lake Photonics, Germany). The ceramic plate was placed in a borosilicate glass vial. Furthermore, the dark current was measured once a day.

### 2.5 Chemometric analysis

The raw reflection spectra of the samples were converted into reflectance spectra by offsetting with the conversion factors for each wavelength and the spectra of the in-house reflection standard and the dark current. Moreover, spectra were converted into Kubelka-Munk units and pseudo-absorbance spectra. Afterwards, spectra were slightly smoothed and treated with several pre-processing techniques, i.e., first or second Savitzky-Golay derivative, standard normal variate transformation (SNV), multiple scatter correction (MSC), orthogonal signal correction (OSC) and two innovative quotient techniques, i.e., “normalized differences” (ND) and “simple ratios” (SR; for more details on ND and SR see Ref. [22]). Each pre-processing technique was combined with one of several regression methods, i.e. partial least squares regression (PLSR), LASSO, ridge, elastic net, least angle regression (LAR), Random Forest (RF), and forward stagewise subset selection combined with PLSR (FS-PLSR). For further information on the chemometric methods, see Ref. [22].

Sample sets 1 and 2 were split into a training set for model calibration and an independent test set for validation at a ratio of 3 to 1. This resulted in 10–27 samples for independent model validation, dependent on the sample set and LOM group (all, pig, cattle), respectively. This procedure was conducted for each of the seven subsample sets (1a–d; 2a–c) and additionally to a combination of spectra from filtrate and filter residues (1e) as well as a combination of spectra from supernatant and pellets (2d). The combination of two spectra, one from the solid and one from the liquid phase, doubles the number of predictor variables, i.e. 4250.

### 2.6 Performance metrics and outlier removal

The performance of the prediction models was evaluated by the squared Pearson coefficient (r^2^), which is a common dimensionless index for expressing the random deviations between observed and predicted values in linear models. The systematic errors, given by the slope and intercept in the linear relationship between observed and predicted values, were not regarded in the model performance evaluation since they can be adjusted.

Two types of outliers were regarded: “Reference outliers” were removed when the reference analysis of a target variable exceeded three times the interquartile range (IQR) from the first or third quartile, respectively. “Prediction outliers” were identified by exceeding three times the RMSEP of the independent test set validation.

## 3 Results and discussion

Table 1, Table 2 provide the statistical distribution of laboratory determined LOM parameters regarding both sample sets 1 and 2. Concentrations are based on both wet weight and dry weight. Both sets comprise pig, cattle and digestate LOM and cover a similar range of nutrient concentrations, DM, and pH values (Fig. 2), common for liquid organic manures. Only P_2_O_5_ and Zn show remarkable discrepancies between the sets. For DM, pH, and macronutrients (wet weight basis), the mean and median are almost identical, implying symmetric distributions. For micronutrients, however, the means are higher than the median, implying a left-skewed distribution. This is also the case for the macronutrient concentration on dry weight basis. In both sample sets, the relative standard deviation (RSD; also coefficient of variation) is lying between 30 and 119 % for DM and all nutrients, with the highest values for Fe and Cu. The distribution of pH values shows a leptokurtic shape with a small RSD of 4–5 %. The samples of both sets represent typical nutrient compositions for the region of Northwest Germany and are therefore suitable as a sample set to investigate the effects of different sample pre-treatment techniques on the predictive performance of spectroscopic measurements.

**Table 1:**
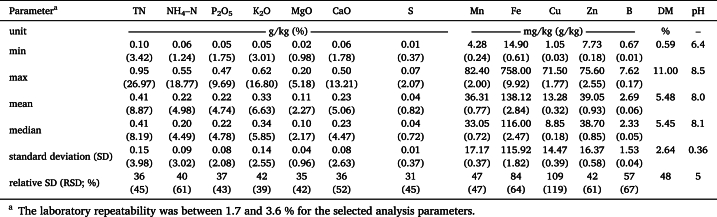
Descriptive statistics of laboratory data on the nutritional composition, DM, and pH of **set 1** with 62 LOM samples (41 pig, 11 cattle, and 10 digestate); nutrient concentrations were determined by standard chemical methods on wet and dry weight basis. Dry weight basis in brackets.

**Table 2:**
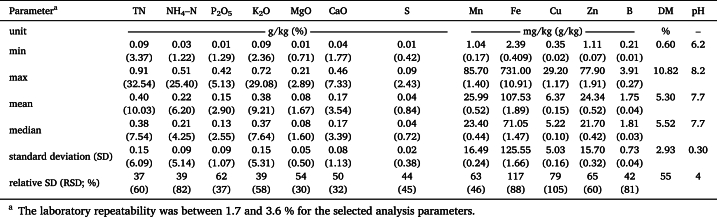
Descriptive statistics of laboratory data on the nutritional composition, DM, and pH of **set 2** with 111 LOM samples (50 pig, 51 cattle, and 10 digestate); nutrient concentrations were determined by standard chemical methods wet and dry weight basis. Dry weight basis in brackets.

**Fig. 2:**
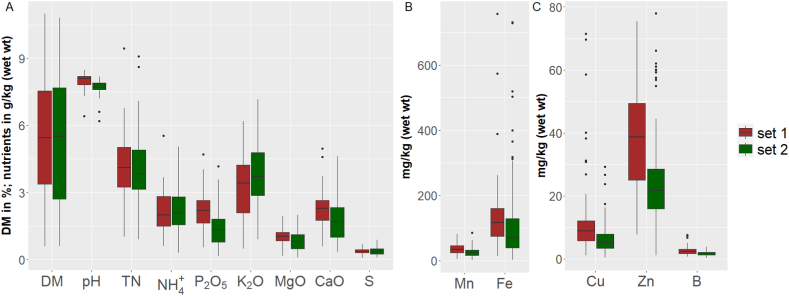
Box-and-whisker plots of laboratory data for DM, pH, and concentrations of 12 nutrients in wet weight for comparing sample set 1 and 2. Plots include interquartile range (IQR) from first and third quartile, median, minimum and maximum and whiskers with a maximum length of 3*IQR for identifying outliers. Note the different scales in subfigures A, B, and C.

Fig. 3, Fig. 4, Fig. 5, Fig. 6 display the performance of the best prediction models within the test sets for different sample pre-treatment techniques. The model's precision is expressed by the squared Pearson coefficient (r^2^), i.e. the random error of the model prediction. In Fig. 3, Fig. 5, models using concentrations on wet weight basis were calculated with the sample spectra of sample set 1 and 2, respectively. In Fig. 4, Fig. 6, the same model calculations were run with the same sample spectra, however, using concentrations on dry weight basis. For detailed regression results, see Supplementary Materials).

**Fig. 3:**
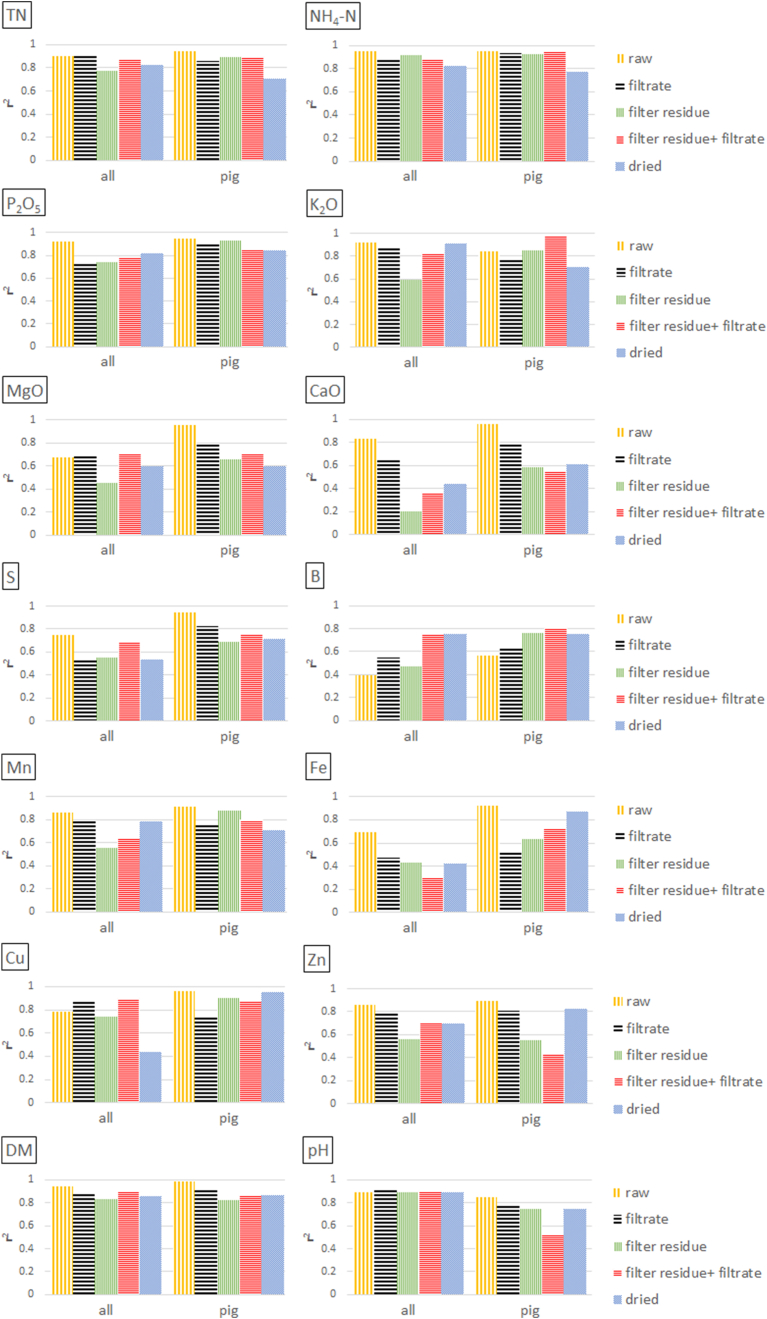
Calibration model performance (r^2^) for LOM properties depending on sample pre-treatment technique (subsamples 1a–1d; see chapter 2.3) and combination of filter residue and filtrate spectra. Concentrations are referring to a **wet weight** basis. Data **set 1** was used, which includes 41 pig, 11 cattle, and 10 digestate LOM samples. Filter residue = wet filter residue representing the solid phase separated by filtration; filter residue + filtrate = combination of spectra from the solid and liquid phase.

**Fig. 4:**
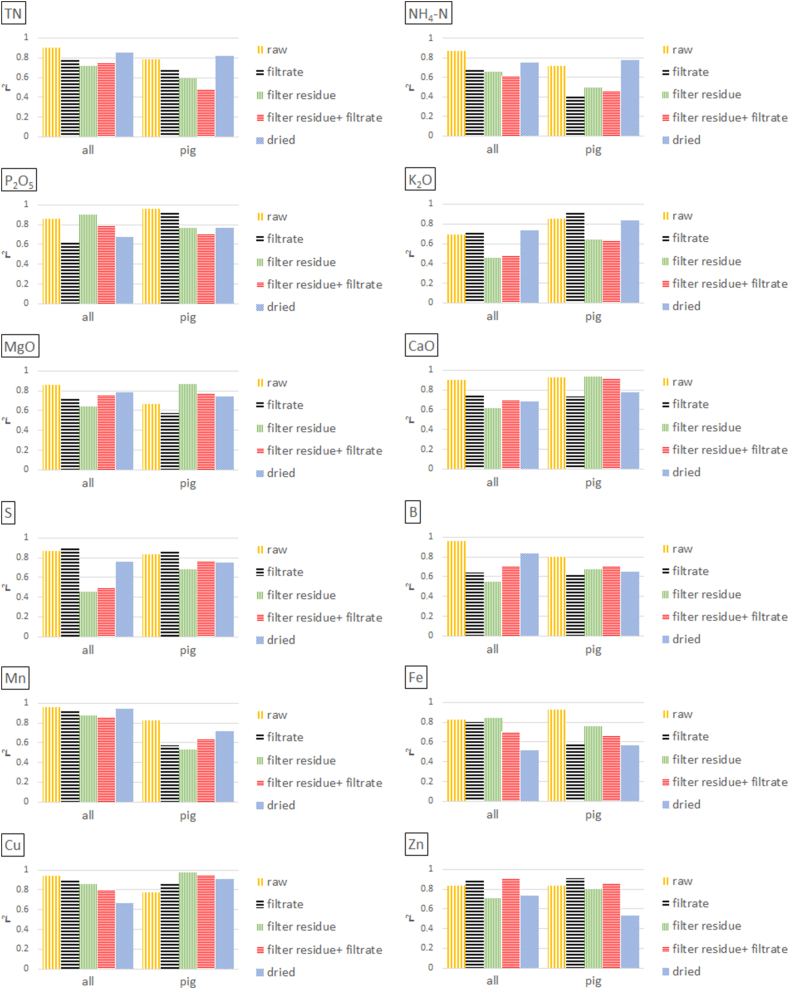
Calibration model performance (r^2^) for LOM properties on **dry weight** basis depending on sample preparation technique (subsamples 1a–1d; see chapter 2.3) and combination of filter residue and filtrate spectra. Data **set 1** was used, which includes 41 pig, 11 cattle, and 10 digestate LOM samples. Filter residue = wet filter residue representing the solid phase separated by filtration; filter residue + filtrate = combination of spectra from the solid and liquid phase.

**Fig. 5:**
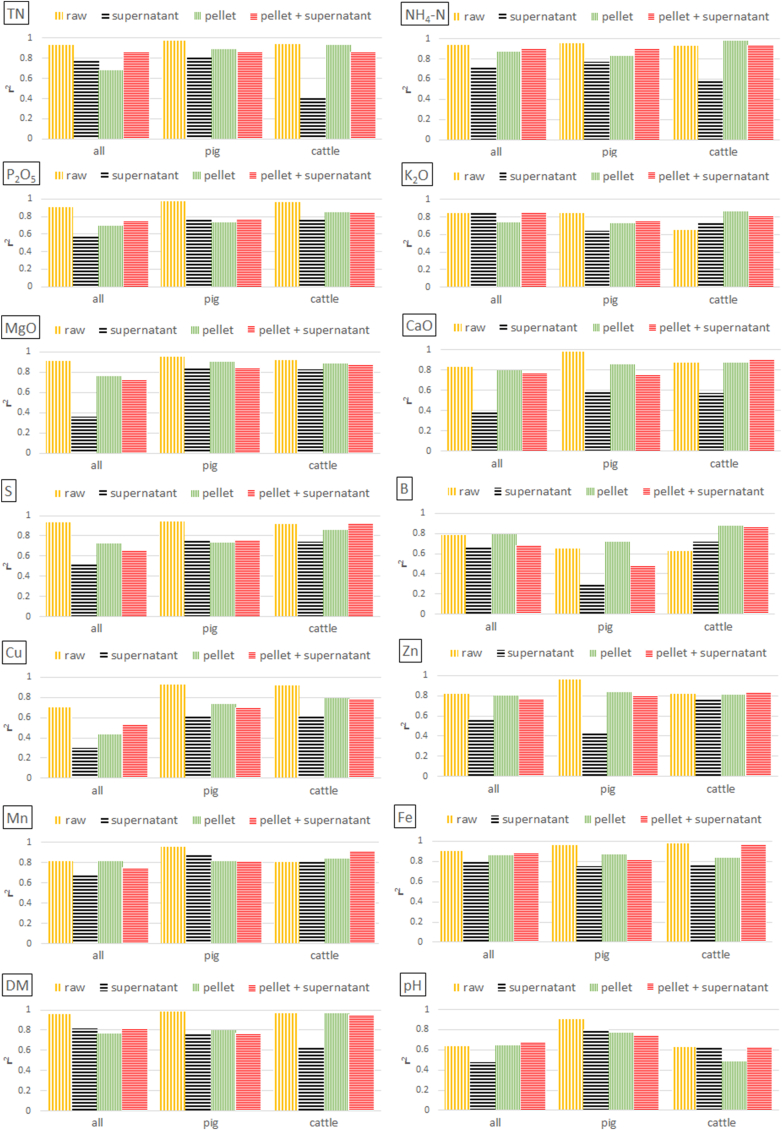
Calibration model performance (r^2^) for LOM properties on **wet weight** basis depending on sample preparation techniques (subsamples 2a–2c; see chapter 2.3) and combination of pellet and supernatant spectra. Data **set 2** was used, which includes 50 pig, 51 cattle, and 10 digestate LOM. Raw = no treatment; pellet = wet pellet representing the solid phase of LOM separated by centrifugation; supernatant = liquid phase separated by centrifugation; pellet + supernatant = spectral combination of the solid and liquid phase.

**Fig. 6:**
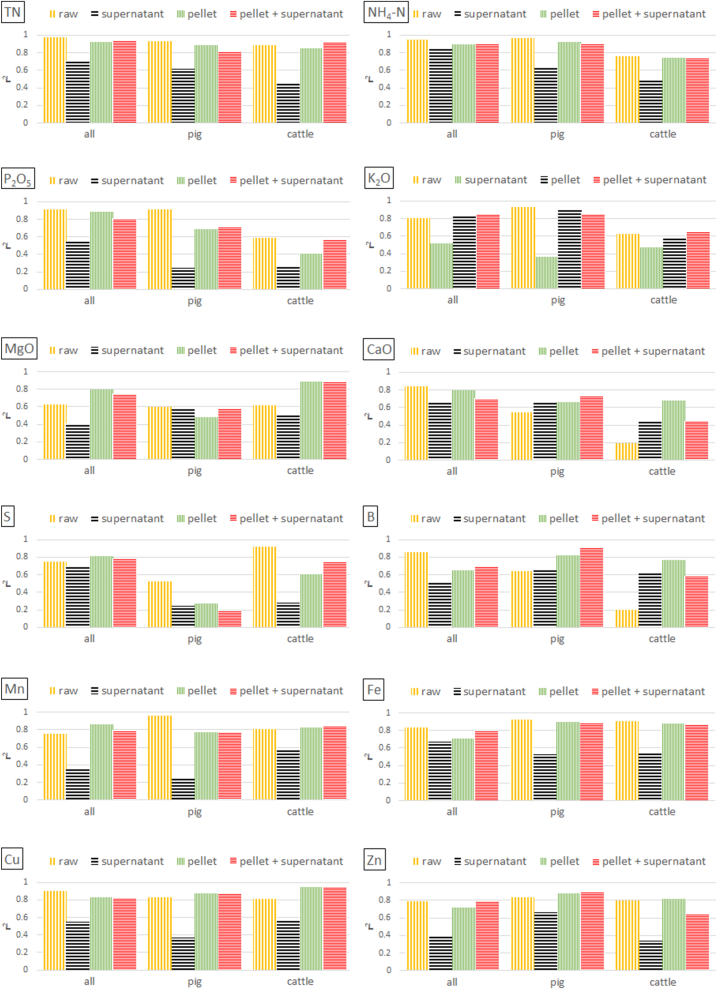
Calibration model performance (r^2^) for LOM properties on **dry weight** basis depending on sample preparation technique (subsamples 2a–2c; see chapter 2.3) and combination of pellet and supernatant spectra. Data **set 2** was used, which includes 50 pig, 51 cattle, and 10 digestate LOM samples. Raw = no treatment; pellet = wet pellet representing the solid phase of LOM separated by centrifugation; supernatant = liquid phase separated by centrifugation; pellet + supernatant = spectral combination of the solid and liquid phase.

In Fig. 3, Fig. 4, the 62 samples (41 pig, 11 cattle, and 10 digestate LOM) of set 1 were prepared by either drying or filtration for separating the solid from the liquid phase. Analyses were conducted using either samples from each species (all) or exclusively pig samples. Samples of digestates and cattle LOM were not analysed separately due to an insufficient number of samples. Overall, it is clearly visible, that neither dried samples nor any phases obtained by filtration (filtrate, filter residue or the spectral combination of both) significantly improved the prediction performance for most target parameters, irrespective of whether they referred to concentrations on wet or wet or dry weight basis. However, some predictions for K, B, Cu, and Zn improved in the following cases:

(i) When using concentrations on wet weight basis and a spectral combination of filtered pig samples and its corresponding filter residues, results for K increased from r^2^ = 0.85 (raw) to 0.97 (Fig. 3 and Appendix Table 1). Similarly, results for K based on dry basis concentrations improved from 0.85 (raw) to 0.91 (filtrate) for pig samples (Fig. 4 and Appendix Table 1). However, when using the total set with all three LOM species (pig + cattle + digestate), this effect was not observed for K, regardless of wet or dry basis concentrations.
(ii) Boron benefited most from sample pre-treatment procedures on wet weight basis, as r^2^ values increased from 0.39 (raw) to 0.48–0.76 for all samples and from 0.57 (raw) to 0.63–0.81 using exclusively pig samples (Fig. 3 and Appendix Table 1). However, this effect was not noticed for B when using concentrations on dry weight basis (Fig. 4 and Appendix Table 1).
(iii) For Cu, results with concentrations on wet weight basis improved in the complete set from r^2^ = 0.79 (raw) to 0.87–0.90 using either filtered samples or a spectral combination of filtered samples and its corresponding filter residues. However, this effect was not obtained for Cu when using exclusively pig samples (Fig. 3 and Appendix Table 1). For the results with reference to concentrations on dry weight basis, it was exactly the opposite. Predictions for pig samples improved from r^2^ = 0.78 (raw) to 0.86–0.97 for one of each preparation technique, whereas this was not the case when using the complete LOM sample set (Fig. 4 and Appendix Table 1).
(iv) Predictions for Zn solely when using dry weight concentrations, namely from r^2^ = 0.84 (raw) to 0.88–0.91 (filtrate or combination of filtrate and filter residue) for the entire sample set (all) and from r^2^ = 0.83 (raw) to 0.91 (filtrate) for the pig set (Fig. 4 and Appendix Table 1).

Finzi et al. [32] investigated the effects of filtering LOM on the chemometric prediction quality. In contrast to the present study, they exclusively analysed the filtrate and not the filter residue. They obtained slightly better predictions for TN and NH_4_–N in the filtrate of a sample set of 12 pig, cattle, and digestate LOM, respectively. These results could not be confirmed in the present study. However, it should be noted that they based their predictions on the chemically determined nutrient concentrations of the filtrate and not on concentrations in the original LOM. Although the concentrations of TN and NH_4_–N in filtered and raw LOM are quite similar, this difference may be a reason for the improved prediction results. Furthermore, calculating concentrations of the original LOM based on predicted filtrate concentrations may not be advisable for other nutrients like P or K, because concentrations might vary significantly between filtered and original samples.

Fig. 5, Fig. 6 show the prediction results for sample set 2 (50 pig, 51 cattle, and 10 digestate LOM) using centrifugation as a pre-treatment procedure. Analyses were conducted using either samples from each species (all) or exclusively pig or cattle samples. Samples of digestates were not analysed separately due to an insufficient number of samples. Although the separation of the liquid and solid phase worked well, also this sample pre-treatment technique did not improve prediction performance noticeable for DM, pH and most nutrients.

Regarding results with concentrations on wet weight basis, an increase in precision was recognized solely in cattle LOM and only for K, B, and Mn when using either supernatants, centrifuged pellets, or a spectral combination of pellets and its corresponding supernatants (Fig. 5 and Appendix Table 1). Regarding dry weight concentrations, predictions similarly improved for B and Mn, but additionally for Mg, Ca, Cu, and Zn, and in one case also for TN and K (Fig. 6 and Appendix Table 1). However, none of these improvements occurred in all three of the tested sample groups (all, pig, cattle). E.g., for Ca, B, and Cu, improvements only occurred for pig or cattle sample analysis groups; for Mn (all), Mg (all, cattle), and Zn (pig). In all the other cases, prediction performance decreased or at least did not significantly improve when analysing spectra of the supernatants, pellets or a combination of both spectra.

It can also be observed that the spectra of the supernatant led to worse results in most cases. One possible reason for this might be the characteristically low reflectance due to a lack of reflecting particles. A reduced centrifuging time or speed resulting in more unsolved particles in the liquid phase might improve prediction results. To the authors’ knowledge, there is no literature on centrifuging experiments with LOM or similar substances, which could serve for comparison.

The specific reasons explaining both the generally deteriorated results (Table 3) and the few improvements of pre-treated LOM (Appendix Table 1) remain unknown. Although the spectral shapes show considerable variations for each pre-treatment (Appendix Figs. 1–7), there seems to be no information gain relevant for improving predictions of the studied LOM properties. However, chemometric analyses for all separated phases were conducted with reference concentrations of the original mixed substances. Probably, predictions for separated phases would increase when referring to chemically determined concentrations of these phases. Nevertheless, farmers are not interested in precise predictions of separated phase concentrations, as they do not split the LOM during field applications. Thus, it is only the total amount of nutrients that is relevant for fertilizing.

**Table 3:** Summary of the different pre-treatment effects on the model performance expressed as the average **r**^**2**^ that includes all LOM properties and samples group calibrations. Maximum underlined.

Set	Raw	Filtrate/supernatant	Filter residue/pellet	Wet + solid fraction	Dried
Set 1 wet wt.	0.86	0.77	0.70	0.75	0.73
Set 1 dry wt.	0.86	0.75	0.71	0.72	0.75
Set 2 wet wt.	0.88	0.66	0.80	0.80	–
Set 2 dry wt.	0.78	0.51	0.78	0.78	–
Average	0.85	0.76 (filter) 0.59 (supernatant)	0.71 (filter residue) 0.79 (pellet)	0.74 (filtering) 0.79 (centrifuging)	0.74

In summary, it can be stated that despite the few improvements in the prediction results, none of the tested sample pre-treatment procedures could justify the labour-intensive preparation steps which could have been conducted for a representative sample of a slurry tank. Nevertheless, it can be concluded from separate tests that intensive homogenization is required immediately before subsampling and right before starting the spectroscopic measurements to guaranty a homogenous subsample and to reverse the segregating process. In the case of dried LOM, this homogenization step can be avoided. However, it should be noted that drying is also an additional time-consuming work step that, based on the available findings, does not improve the prediction performance, neither on wet nor on dry basis concentrations. There are some publications dealing with chemometric analyses of dried manure spectra [24,[28], [29], [30], [31]]. However, these studies did not compare predictions of dried samples with those of raw LOM. Moreover, the reported performance metrics were not outstanding compared to similar publications working with liquid organic manures [25].

## 4 Conclusions

In an exhaustive study of two large samples sets including different LOM (pig and cattle manures, biogas digestates), samples were pre-treated with three different sample preparation techniques (drying, filtering, centrifuging) before analysing for macronutrients, micronutrients, dry matter and pH by Vis-NIR spectrometry. Compared to the analysis of the unseparated, original LOM, none of these sample pre-treatment procedures significantly improved prediction results, independent from LOM type, LOM property, or concentrations based either on wet or dry weight. Thus, there is no need for time-consuming procedures separating the liquid and solid phase of LOM for optical spectrometry. However, an important prerequisite for optimal prediction results is a sufficient homogenization of LOM. Nevertheless, optical spectrometry has the potential to become an appropriate measurement technique to directly quantify nutrients of LOM at farm level, whether in the storage tank, while filling a slurry tanker, or during application in the field [22].

## Data availability statement

Corresponding data is available on request.

## CRediT authorship contribution statement

**Michael Horf:** Writing – review & editing, Writing – original draft, Visualization, Validation, Software, Resources, Methodology, Investigation, Formal analysis, Data curation, Conceptualization. **Robin Gebbers:** Writing – review & editing, Supervision, Project administration, Funding acquisition, Conceptualization. **Hans-Werner Olfs:** Writing – review & editing, Project administration, Funding acquisition. **Sebastian Vogel:** Writing – review & editing, Supervision, Project administration.

## Declaration of competing interest

The authors declare that they have no known competing financial interests or personal relationships that could have appeared to influence the work reported in this paper.

## Figures and Tables

**Table 1:** Summary of improvements expressed as r^2^ regarding the different pre-treatments in reference to no pre-treatment (raw). Improvements were registered when r^2^ increased at least more than 0.03 units. Here, x means no improvement and – means no measurements.

Set	Subset	Property	Raw	Filtrate/supernatant	Filter residue/pellet	Wet + solid fraction	Dried
**Set 1 wet wt.**	all	B Cu	0.39 0.79	0.55 0.87	0.48 x	0.76 0.90	0.76 x
	pig	K B	0.85 0.57	x 0.63	x 0.76	0.97 0.81	x 0.75
**Set 1 dry wt.**	all	Zn	0.84	0.88	x	0.91	x
	pig	K Cu Zn	0.85 0.77 0.83	0.91 0.86 0.91	x 0.97 x	x 0.96 x	x 0.91 x
**Set 2 wet wt.**	all	x	x	x	x	x	–
	pig	x	x	x	x	x	–
	cattle	K B Mn	0.65 0.63 0.81	0.73 0.71 x	0.86 0.88 0.84	0.82 0.87 0.92	–
**Set 2 dry wt.**	all	K Mg Mn	0.81 0.62 0.75	x x x	x 0.80 0.87	0.85 0.74 0.80	–
	pig	Ca B Cu Zn	0.55 0.64 0.83 0.84	0.65 x x x	0.66 0.82 0.87 0.88	0.74 0.91 0.87 0.90	–
	cattle	TN Mg Ca B Cu	0.88 0.62 0.19 0.21 0.81	x x 0.45 0.62 x	x 0.89 0.68 0.77 0.95	0.93 0.89 0.44 0.59 0.96	–

**Fig. 1:**
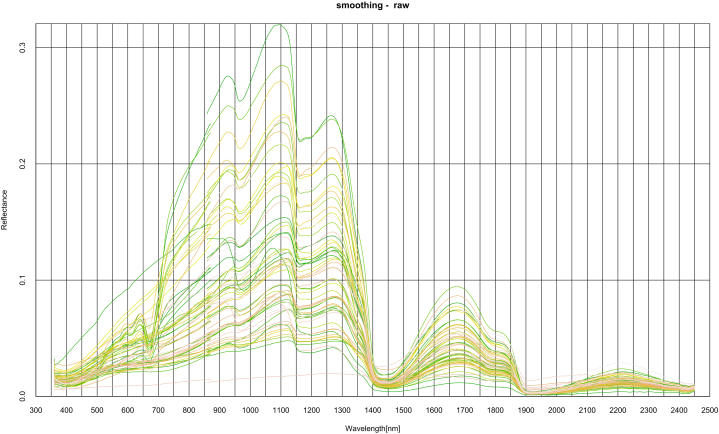
Smoothed reflectance spectra of set 1a - 62 untreated samples (raw) - different colours only for better distinction between spectral lines.

**Fig. 2:**
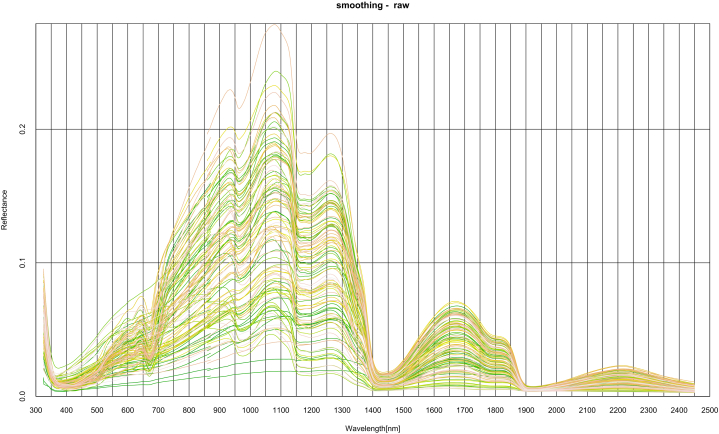
Smoothed reflectance spectra of set 2a - 111 untreated samples (raw) - different colours only for better distinction between spectral lines.

**Fig. 3:**
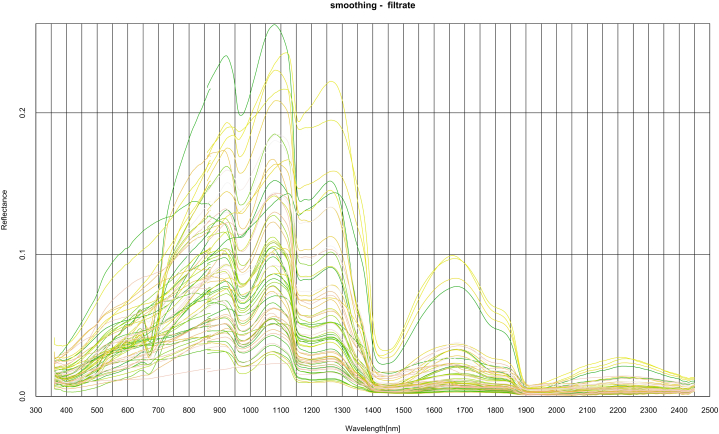
Smoothed reflectance spectra of set 1b - 62 filtered samples (filtrate) - different colours only for better distinction between spectral lines.

**Fig. 4:**
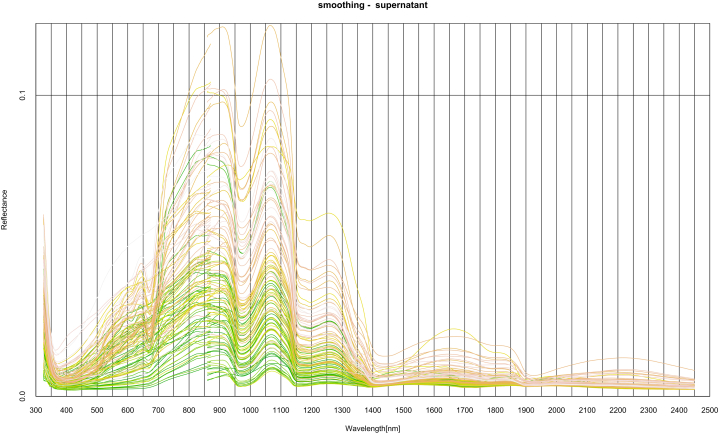
Smoothed reflectance spectra of set 2b - 111 centrifuged samples (supernatant) - different colours only for better distinction between spectral lines.

**Fig. 5:**
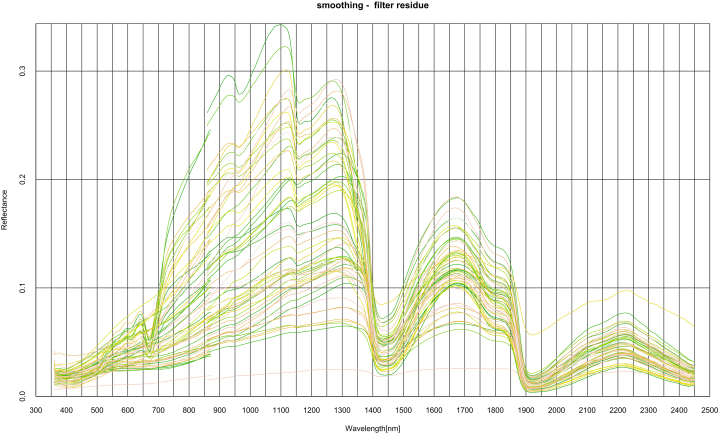
Smoothed reflectance spectra of set 1c- 62 filtered samples (filter residues) - different colours only for better distinction between spectral lines.

**Fig. 6:**
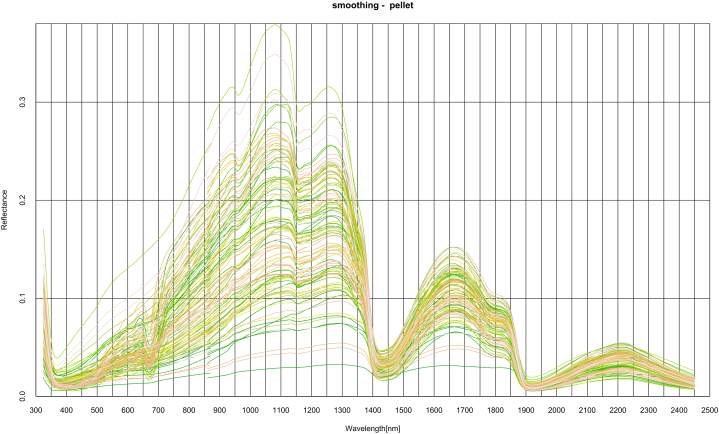
Smoothed reflectance spectra of set 2c - 111 centrifuged samples (pellets) - different colours only for better distinction between spectral lines.

**Fig. 7:**
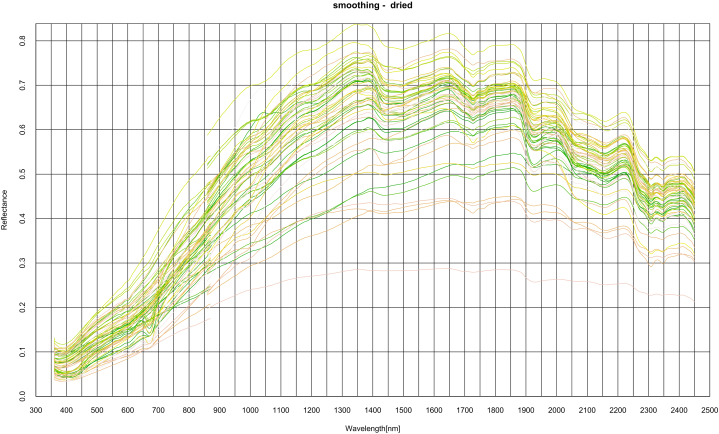
Smoothed reflectance spectra of set 1d - 62 dried samples - different colours only for better distinction between spectral lines.

## References

[1] Ben Hassen T., El Bilali H. (2022). Impacts of the Russia-Ukraine war on global food security: towards more sustainable and resilient food systems?. Foods.

[2] Baştabak B., Koçar G. (2020). A review of the biogas digestate in agricultural framework. J. Mater. Cycles Waste Manag..

[3] Loss A., Da Rosa Couto R., Brunetto G., Da Veiga M., Toselli M., Baldi E. (2019). Animal manure as fertilizer: changes in soil attributes, productivity and food composition. Int. J. Regul. Govern..

[4] Prado J., Ribeiro H., Alvarenga P., Fangueiro D. (2022). A step towards the production of manure-based fertilizers: disclosing the effects of animal species and slurry treatment on their nutrients content and availability. J. Clean. Prod..

[5] Menegat S., Ledo A., Tirado R. (2022). Author Correction: greenhouse gas emissions from global production and use of nitrogen synthetic fertilisers in agriculture. Sci. Rep..

[6] He Z., Ding B., Pei S., Cao H., Liang J., Li Z. (2023). The impact of organic fertilizer replacement on greenhouse gas emissions and its influencing factors. Sci. Total Environ..

[7] Ray R.L., Griffin R.W., Fares A., Elhassan A., Awal R., Woldesenbet S., Risch E. (2020). Soil CO2 emission in response to organic amendments, temperature, and rainfall. Sci. Rep..

[8] Rayne N., Aula L. (2020). Livestock manure and the impacts on soil health: a review. Soil Systems.

[9] Foged H., Flotats X., Bonmatí A., Palatsi J., Magrí A., Schelde K. (2011).

[10] Zhang H., Schroder J., He Z., Zhang H. (2014). Applied Manure and Nutrient Chemistry for Sustainable Agriculture and Environment.

[11] Waldrip H.M., Pagliari P.H., He Z. (2020).

[12] Saeys W., Watté R., Postelmans A. (2019). Proceedings of the International Fertilizer Society (Ifs).

[13] Saeys W., Darius P., Ramon H. (2004). Potential for on-site analysis of hog manure using a visual and near infrared diode array reflectance spectrometer. J. Near Infrared Spectrosc..

[14] Ahdb - Agriculture, Horticulture Development BoardSiskin Parkway East (2023).

[15] De Ferrari G., Gallina P.M., Cabassi G., Bechini L., Maggiore T. (2005). NIR 2005- NIR in Action. Making a Difference. Near Infrared Spectroscopy. Proceedings of the 12th International Conference 2005.

[16] Saeys W. (2006).

[17] Saeys W., Mouazen A.M., Ramon H. (2005). Potential for onsite and online analysis of pig manure using visible and near infrared reflectance spectroscopy. Biosyst. Eng..

[18] Park J., Kang T., Heo Y., Lee K., Kim K., Lee K., Yoon C. (2020). Evaluation of short-term exposure levels on ammonia and hydrogen sulfide during manure-handling processes at livestock farms. Saf. Health Work.

[19] Piepel M.-F., Dittert K., Olfs H.-W. (2022). Evaluation of physicochemical on-farm quick tests for estimating nutrient concentrations in pig slurry and development of an application for mobile devices. Agronomy.

[20] Piepel M.-F., Olfs H.-W. (2023). Development of a physicochemical test kit for on-farm measurement of nutrients in liquid organic manures. Agriculture.

[21] Sefeedpari P., Pudełko R., Jędrejek A., Kozak M., Borzęcka M. (2020). To what extent is manure produced, distributed, and potentially available for bioenergy? A step toward stimulating circular bio-economy in Poland. Energies.

[22] Horf M., Gebbers R., Olfs H.-W., Vogel S. (2024). Determining nutrients, dry matter, and pH of liquid organic manures using visual and near-infrared spectrometry. Sci. Total Environ..

[23] Kumar N., Bansal A., Sarma G.S., Rawal R.K. (2014). Chemometrics tools used in analytical chemistry: an overview. Talanta.

[24] Asai T., Shimizu S., Koga T., Sato M. (1993). Quick determination of total nitrogen, total carbon and crude ash in cattle manure using near infrared reflectance spectroscopy. Jpn. J. Soil Sci. Plant Nutr..

[25] Horf M., Vogel S., Drücker H., Gebbers R., Olfs H.-W. (2022). Optical spectrometry to determine nutrient concentrations and other physicochemical parameters in liquid organic manures: a review. Agronomy.

[26] Derikx P., van de Kooi B., Heskamp H., Rozijn M. (2021).

[27] Saeys W., Nguyen Do Trong N., van Beers R., Nicolaï B.M. (2019). Multivariate calibration of spectroscopic sensors for postharvest quality evaluation: a review. Postharvest Biol. Technol..

[28] Althaus B., Papke G., Sundrum A. (2013). Technical note: use of near infrared reflectance spectroscopy to assess nitrogen and carbon fractions in dairy cow feces. Anim. Feed Sci. Technol..

[29] Malley D.F., McClure C., Martin P.D., Buckley K., McCaughey W.P. (2005). Compositional analysis of cattle manure during composting using a field‐portable near‐infrared spectrometer. Commun. Soil Sci. Plant Anal..

[30] Reeves J.B. (2001). Near- versus mid-infrared diffuse reflectance spectroscopy for determination of minerals in dried poultry manure. Poultry Sci..

[31] Dong Y., Chen Y., Zhu D., Li Y., Xu C., Bai W., Wang Y., Li Q., Li D., Liu Y., Chen Y. (2011). Computer and Computing Technologies in Agriculture IV.

[32] Finzi A., Oberti R., Negri A.S., Perazzolo F., Cocolo G., Tambone F., Cabassi G., Provolo G. (2015). Effects of measurement technique and sample preparation on NIR spectroscopy analysis of livestock slurry and digestates. Biosyst. Eng..

